# Ouabain affects cell migration via Na,K-ATPase-p130cas and via nucleus-centrosome association

**DOI:** 10.1371/journal.pone.0183343

**Published:** 2017-08-17

**Authors:** Young Ou, Chen Xuan Pan, Jeremy Zuo, Frans A. van der Hoorn

**Affiliations:** Department of Biochemistry & Molecular Biology, Cumming School of Medicine, University of Calgary, Calgary, Alberta, Canada; Universidade Federal do Rio de Janeiro, BRAZIL

## Abstract

Na,K-ATPase is a membrane protein that catalyzes ATP to maintain transmembrane sodium and potassium gradients. In addition, Na,K-ATPase also acts as a signal-transducing receptor for cardiotonic steroids such as ouabain and activates a number of signalling pathways. Several studies report that ouabain affects cell migration. Here we used ouabain at concentrations far below those required to block Na,K-ATPase pump activity and show that it significantly reduced RPE cell migration through two mechanisms. It causes dephosphorylation of a 130 kD protein, which we identify as p130cas. Src is involved, because Src inhibitors, but not inhibitors of other kinases tested, caused a similar reduction in p130cas phosphorylation and ouabain increased the association of Na,K-ATPase and Src. Knockdown of p130cas by siRNA reduced cell migration. Unexpectedly, ouabain induced separation of nucleus and centrosome, also leading to a block in cell migration. Inhibitor and siRNA experiments show that this effect is mediated by ERK1,2. This is the first report showing that ouabain can regulate cell migration by affecting nucleus-centrosome association.

## Introduction

Na,K-ATPase is a membrane protein that catalyzes ATP to maintain transmembrane sodium and potassium gradients [1]. During each functional cycle, it pumps three sodium ions out and transports two potassium ions into the cell for each hydrolyzed molecule of ATP. The enzyme consists of two nonconvalently linked subunits: the α-subunit contains the ATP catalytic domain and the β-subunit may facilitate the insertion of the α-subunit into the correct location at the cell membrane [2,3].

Ouabain, derived from plants, has been used to treat heart disease for more than a century. Ouabain binds, with high affinity and specificity, to the extracellular domain of the α-subunit of Na,K-ATPase. The binding inhibits the enzyme’s function, thereby altering the transmembrane electrochemical potential of the cell. In addition to altering the pump activity, ouabain binding to Na,K-ATPase was shown to also trigger signaling pathways including IP3R/calcium and Src pathways [4–8]. Specifically, Na,K-ATPase interacts via its the N-terminal domain with the SH2 and kinase domains of Src [9,10]. It is believed that binding of ouabain to Na,K-ATPase releases the kinase domain of Src, which transactivates the epidermal growth factor receptor (EGFR) and in turn activates the MAPK pathway [10]. Inhibition of the pump activity requires ouabain at micromolar (1–10 μM) concentration, but ouabain can trigger signaling pathways at picomolar to nanomolar concentrations (for review see [11]. Different Na,K-ATPase isoforms can have different sensitivity to ouabain. It is estimated that at nanomolar concentrations ouabain binds only 1 per 104 Na,K-ATPase molecules [12].

In preliminary studies, we observed that ouabain at nanomolar concentrations can cause a block in cell migration in several cell lines, including RPE cells. This is in agreement with recent reports showing that ouabain can affect cell migration [13,14]. The predominant Na,K-ATPase subunits expressed in RPE cells are the α1 and β1 subunit [15], but α2 and β2 subunits were also described [16].

Here, we explored the signaling pathway(s) in RPE cells that may be involved in this phenomenon. Since the ouabain-src connection had been established previously, we first focused on possible phosphorylation changes. Ouabain treatment significantly reduced tyrosine-phosphorylation of a 130 kDa protein, which we identified as p130cas. Specific RNAi of p130cas confirmed its role in cell migration. p130cas was shown previously to be a critical signaling node implicated in the regulation of actin polymerization and cell migration [17,18]. Examination of cells treated with ouabain at nanomolar concentrations showed actin fiber disruption. Using kinase inhibitors, we found a link between ouabain, p130cas and src. Second, we observed separation of nucleus and centrosome upon nanomolar ouabain treatment of cells. We had previously shown using a system of ATP and hypoxia that such separation causes a block in cell migration [19]. RNAi and kinase inhibitors suggested that ERK is critically involved in this pathway. Thus, we identified two signaling pathways activated by ouabain that control cell migration.

## Materials and methods

### Chemicals and antibodies

Ouabain and phalloidin were purchased from Sigma-Aldrich (St. Luis, MO, USA). The EGFR inhibitor Iressa was purchased from Tocris (Bristol, UK). Src inhibitor AZD0530, MEK inhibitor PD0325901, and p38MAPK inhibitor VX702 were purchased from Selleckchem (Houston, USA). Src inhibitor PP2 and PI3K inhibitor TGX221 were purchased from EMD Millipore (Darmstadt, Germany). Anti-Src antibody and anti-phospho Y416-Src antibody were a gift from Dr. Don Fujita at the University of Calgary. Anti-p130 antibody was purchased from Abcam (Cambridge, MA). Rabbit polyclonal anti-Na,K-ATPase and mouse monoclonal antibody 4G10 were purchased from EMD Millipore. Anti-β-actin antibody was purchased from Sigma-Aldrich. Anti-ninein antibody was characterized previously[20]. HRP-conjugated secondary antibody and Cy3- and Alex488-labeled secondary antibodies were purchased from Jackson ImmunoResearch (West Grove, PA) and Molecular Probes (Eugene, OR), respectively. Anti-ERK antibody was purchased from Cell Signaling Technologies (Danvers, MA) and Fluorescent-labeled ouabain was purchased from ThermoFisher Scientific (Waltham, MA).

### Cell culture and drug treatment

Human retinal pigmented epithelia (RPE) cells (ATCC, Manassas, VA), HS86 foreskin fibroblasts [21] and U87 glioma cells (ATCC) were grown in DMEM (Gibco/BRL) supplemented with 10% fetal calf serum. For preparation of log phase cells, cells were collected by trypsin/EDTA treatment (Gibco/BRL) and re-grown overnight in fresh medium. Drugs were applied to log phase cells at the concentration indicated in the text and cell extracts collected by lysis on plates. For ouabain labeling dynamics, fluorescent-labeled ouabain was diluted to 1 μM in Hanks solution (Invitrogen) and incubated with cells for the time indicated in the text.

### Immunoprecipitations

Immunoprecipitations were performed as described previously [21]. Briefly, cell extracts were prepared in lysis buffer (10 mM Tris pH 7.4, 150 mM NaCl, 10 mM KCl, 1 mM EDTA pH 8.0, 0.5% DOC, 0.5% Tween-20, 0.5% NP-40, 1x proteinase inhibitor cocktail (Roche)). Protein extracts were pre-cleared by mixing with 20 μl protein G Sepharose beads and incubated overnight at 4°C with either anti-HA antibody or anti-GFP antibody as stated in the text. Following incubation, beads were precipitated and washed three times with cell lysis buffer. After a final wash, proteins were eluted from the beads with SDS-sample buffer, and analyzed by gel electrophoresis and western blotting. All experiments were reproducible.

### Immuno-affinity purification and mass spectrometry analysis

Cells collected from 10 dishes (150 mm) were solubilized in 5 ml of TBS buffer (50 mM Tris, pH7.5, 150 mM NaCl, 1% NP-40, 0.5% Triton X-100 and 1X protease inhibitor cocktail). After a brief centrifugation, extracts were incubated with 500 μl of 4G10 antibody-conjugated beads (EMD Millipore) overnight at 4°C with constant rotation. The beads were washed three times with 3 ml of TBS buffer and proteins were eluted with 500 μl of elution buffer (0.1 M glycine, pH2.5). Eluates were lyophilized, resuspended in 50 μl of 1x SDS-sample buffer, and separated by 10% SDS-PAGE. After electrophoresis, the gel area around 130 kDa was cut out and gel slices were subjected to In-Gel LC-MS/MS mass spectrometry analysis performed at the University of Calgary Mass Spectrometry facility.

### siRNA experiments

All of the following siRNA products were purchased from Qiagen: negative control (scrambled) siRNA Cat # SI03650325; p130cas Cat# SI02757734 and SI02757741; MAPK1/3 (ERK1/2) Cat# SI00605990 and Cat # SI00605997. The siRNA products were transfected using RNAi-Max (Invitrogen-Thermo Fisher Scientific, MA, USA) and cells were cultured for 72 hrs. Cells were fixed with cold methanol and analyzed by immunofluorescence microscopy or were collected for analysis by semi-quantitative RT-PCR. The two ERK1/2 siRNA oligos were combined and used at the concentration of 25 nM final concentration.

### Western blotting

Western blot analysis was carried out as follows: protein extracts were boiled in loading buffer, separated by 10% SDS-PAGE, electrophoretically transferred onto a polyvinylidene fluoride membrane (Amersham Biosciences), blocked overnight at 4°C in blocking buffer (54 mM Tris, pH 7.5, 150 mM NaCl, 0.05% Nonidet P-40, 0.05% Tween 20, 5% nonfat dry milk) and analyzed using primary antibodies followed by HRP-conjugated secondary antibody. Prestained Protein Ladder SM0671 (Fermentas) was used as a size marker. LumiGLO substrate (Kirkegaard & Perry Laboratories, Inc.) was used to develop the blot. Luminescence was captured using Amersham hyperfilm ECL films (Amersham-GE Healthcare Bio-Sciences, PA, USA).

### Immunofluorescence microscopy

Immunofluorescence microscopy was performed as described previously [21]. Briefly, cells were fixed in cold methanol and stained with primary and secondary antibodies described in the text at a concentration recommended by the manufacturer. Images were obtained using a Zeiss microscope with 40x objective in conjunction with Zeiss software AxioVision Rel.4.9.1.

### Cell migration assays

The cell scratch assay was done using established procedures [22]. Briefly, cells were grown to confluence. Then, a micropipette tip was used to make a scratch in the monolayer. Images of the damaged area were taken immediately and after 7.5 and 48 hrs. The width of the scratch gaps was measured using a Zeiss microscope and Axiovert software Measurement-Length function. To measure the effect of ouabain on cell migration, cells were treated with 50 nM ouabain.

The Transwell assay was carried out as follows: RPE cells were grown in DMEM medium/10% FBS. At 70% confluence, cells were treated for 1 day with either fresh medium (control) or medium that contains 5 μM ouabain. Next, cells were trypsinized and resuspended in 1.5 ml DMEM without serum. 5 x 10^5^ cells from each treatment were loaded into the upper chambers of a Transwell kit (Corning Inc, Corning, NY, USA). The lower chamber was filled with 2.0 ml DMEM/10% FBS. After 1.5 days of incubation cells that had migrated and adhered to the lower chamber substratum were imaged and counted.

### Statistical analysis

Data are presented as the mean value or mean +/- s.d. (standard deviation) of at least three independent experiments. Student’s t-test was used to determine P value. P values less than 0.05 are considered to be significant.

## Results

### Ouabain reduces tyrosine phosphorylation of a 130 kDa protein

**Fig 1:**
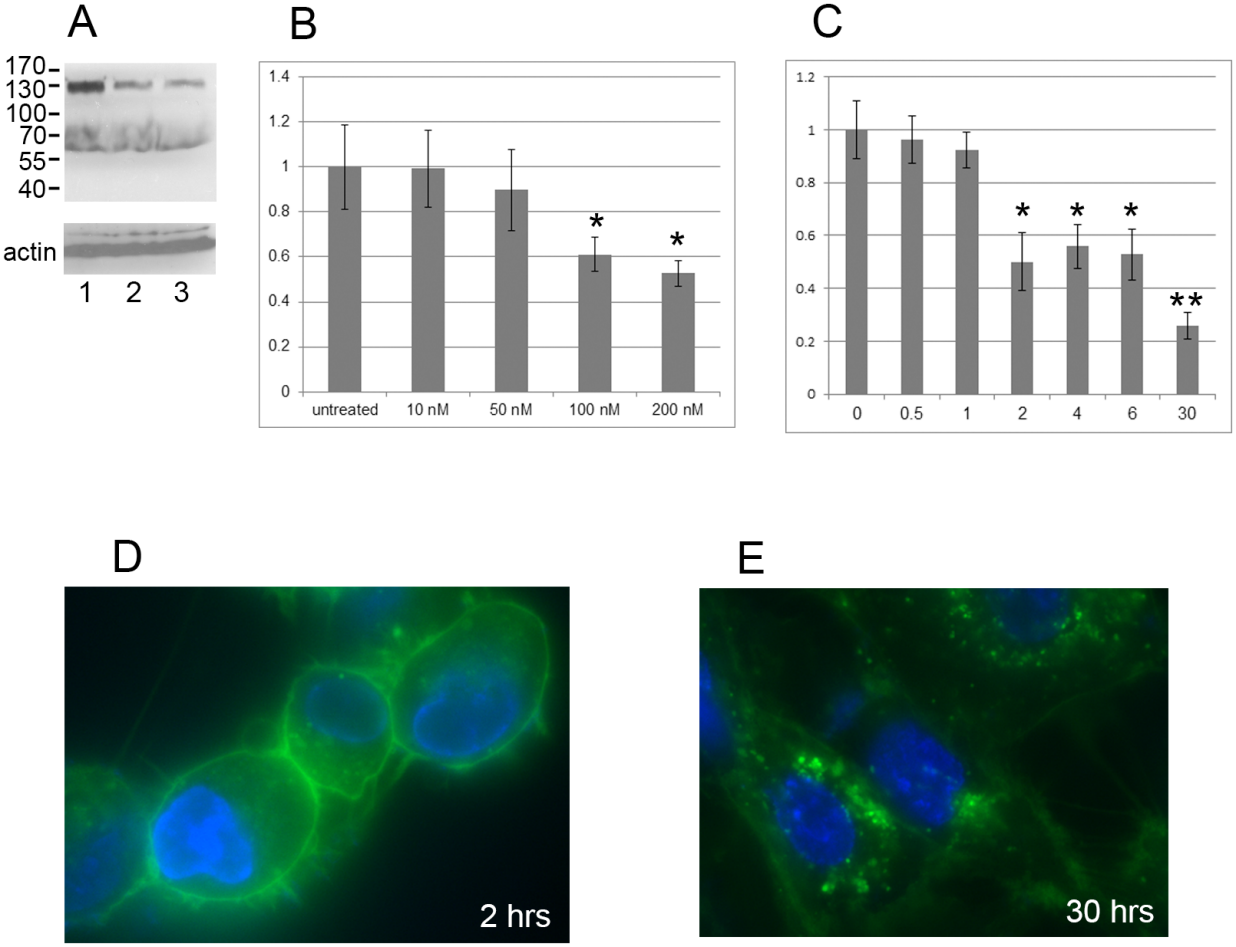
Ouabain causes dephosphorylation of a 130 kDa protein. (A) Cells were treated with ouabain for 24 hrs, collected and analysed by western blotting using 4G10 monoclonal antibody (upper panel) and anti-actin antibody as a loading control (lower panel). Lane 1, untreated control; lane 2, ouabain at 100 nM; lane 3, ouabain at 500 nM. (B) Ouabain dose response curve. Cells were treated with indicated concentrations of ouabain and analyzed by western blotting using 4G10 monoclonal antibody and anti-actin antibody. Quantitation of the amount of tyrosine phosphorylated 130 kDa protein was done using NIH Image J. The diagram shows the change of the treated sample with respect to untreated control (set at 1). The difference between untreated and 100 and 200 nM is statistically significant (P<0.05) (indicated by *). (C) Ouabain time course analysis. Cells were treated with 100 nM ouabain for indicated times (hrs) and analyzed as above. Quantitation of the amount of tyrosine phosphorylated 130 kDa protein was done using NIH Image J. The diagram shows the change after treatments with the zero-time point arbitrarily set at 1. The difference between 0 hrs and 2–6 hrs is statistically significant (P<0.05; indicated by *) and the difference between 30 hrs and earlier time points is also statistically significant (P<0.05; indicated by **). (D) and (E) Fluorescent-labeled ouabain was incubated with RPE cells and images were taken at 2 hrs (D) and 30 hrs (E). Green represents fluorescent-labeled ouabain and blue represents DAPI-stained DNA.

Preliminary experiments had shown that ouabain treatment can affect cell migration. To investigate involved pathways, we used three human cell types: RPE cells, HS68 primary foreskin fibroblasts, and U87 glioma cells. Ouabain was added to cells at final concentrations of 100 nM and 500 nM and proteins were analyzed by western blotting using 4G10, a well-characterized monoclonal antibody against phospho-tyrosine [23]. As shown in Fig 1A ouabain treatment of RPE cells reduced Tyr phosphorylation of a 130 kDa protein. Since the three cell types exhibited the same result after ouabain treatment (not shown), the following experiments were done using RPE cells unless otherwise indicated.

First, we analyzed if the change in Tyr phosphorylation level of the 130 kDa protein by ouabain is concentration dependent. The results shown in Fig 1B illustrate that the Tyr phosphorylation status of the 130 kDa protein showed a response to ouabain with a gradual decrease that became statistically significant at 100 and 200 nM ouabain. Next, we measured the time course for ouabain to mediate this effect using 100 nM ouabain. Fig 1C shows that ouabain significantly affected Tyr phosphorylation of the 130 kDa protein as early as 2 hrs post treatment, and that this reduction continued over time. The ouabain effect was reversible. These results show that Na,K-ATPase controls Tyr phosphorylation of a 130 kDa protein in a dose- and time-dependent manner. The use of fluorescently-labeled ouabain showed that at the 2 hr timepoint ouabain is predominantly staining the cell surface, as expected (Fig 1D). Interestingly, after 30 hrs ouabain can be seen internalized in the cells with little ouabain remaining at the cell surface (Fig 1E). We next used this system to investigate mechanisms of action of ouabain.

### Identification of the 130 kDa protein as p130cas

**Fig 2:**
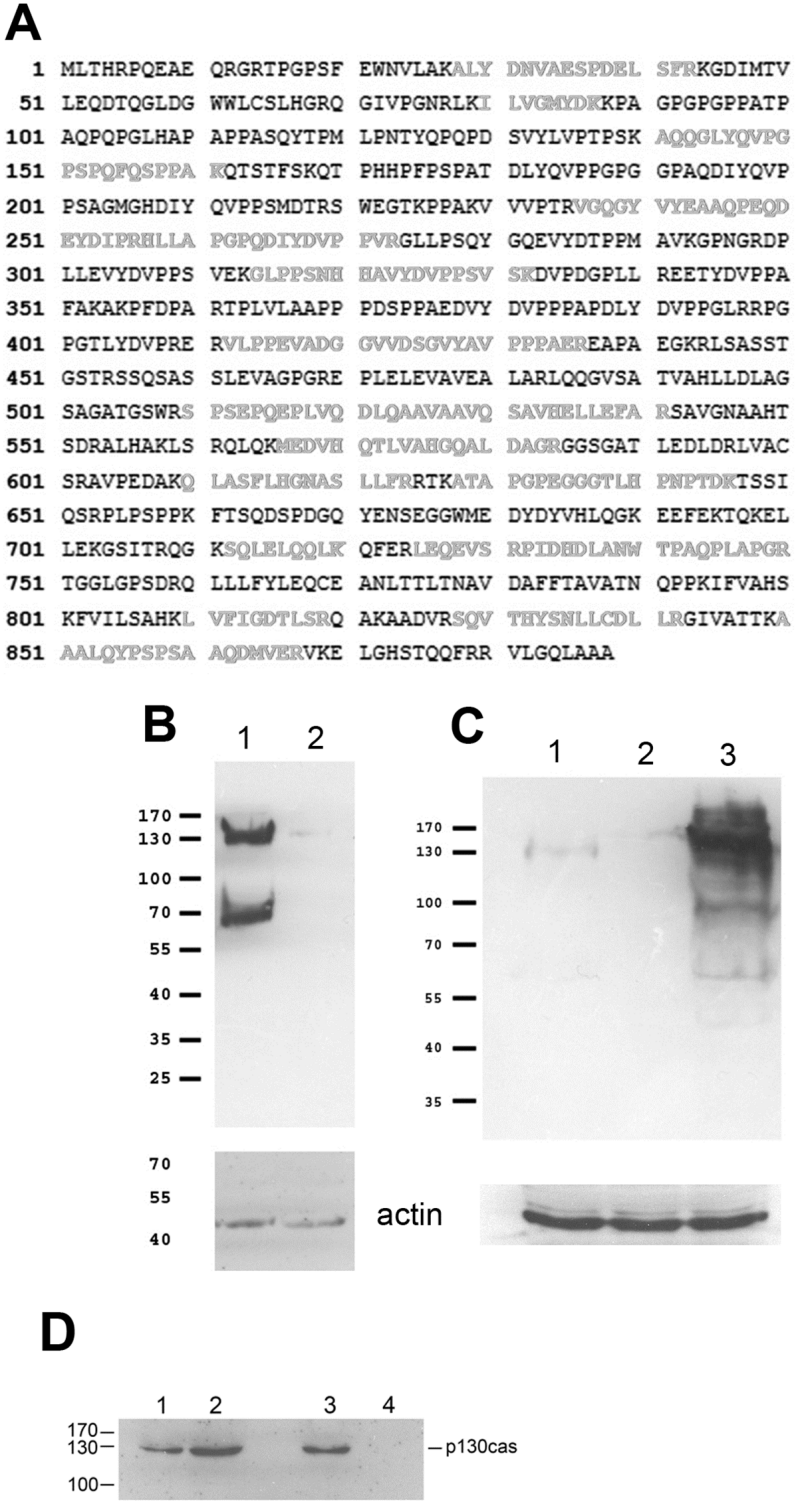
Identification of the 130 kDa protein as p130cas. (A) The 130 kD protein was immunoprecipitated and gel-purified for mass spectrometry analysis as described in the Materials & Methods. MASCOTT analysis of mass spectrometry data shows that the 130 kDa protein is p130cas. Amino acids indicated in grey represent peptides detected by mass spectrometry that are present in p130cas. (B) Since p130cas phosphorylation is known to be sensitive to cell detachment we analyzed if the 130 kDa protein shows the same characteristic: cells were lysed directly on plates (lane 1) or were lysed after scraping the cells off plates (lane 2) and analysed for 130 kDa Tyr phosphorylation using western blotting. Upper panel, 4G10 monoclonal antibody; lower panel, anti-actin antibody. (C) p130cas phosphorylation is dramatically increased by H_2_O_2_. We studied if the p130 protein responds similarly. Tyr phosphorylation of the 130 kDa protein was compared between untreated cells grown normally on plates (lane 1), cells detached by scraping off plates (lane 2), and cells treated with 0.03% H_2_O_2_ for 10 min (lane 3). Upper panel, 4G10 monoclonal antibody; lower panel, anti-actin antibody. Note that the upper panel in C) was exposed for a much shorter time than the panel shown in B), to avoid strong overexposure in lane 3. (D) To further establish p130 as p130cas we carried out protein analysis of RPE cell extracts by western blotting assays using anti-p130cas antibody directly or after immunoprecipitation assays using 4G10 monoclonal antibody. Lanes 1 and 3, total cell extracts; lane 2, 4G10 immunoprecipitate; lane 4, non-specific mouse IgG immunoprecipitate.

To identify the 130 kDa protein we performed an affinity purification assay. RPE cells were lysed and extracts were incubated with 4G10-conjugated agarose beads. Proteins bound to the beads were fractionated by SDS-PAGE and proteins eluted from gel slices were analyzed by mass spectrometry. The results indicated that > 15 peptides derived from the isolated 130 kDa protein match the sequence of p130cas, with a protein sequence coverage of 32% (Fig 2A).

We next studied if p130 protein exhibits well-documented characteristics of p130cas. p130cas is a scaffold molecule that interacts with the focal adhesion kinase (FAK)/Src dual kinase complex [9,24,25]. p130cas acts as a signaling node participating in many intracellular signaling events including integrin signalling [26–28]: p130cas is sensitive to the state of cell adhesion [25,29,30]. To study if the 130 kDa protein exhibits this characteristic, we analyzed if Tyr phosphorylation of the 130 kDa protein is sensitive to cell adhesion by comparing 130 kDa Tyr phosphorylation before and after detachment from culture plates. The results show that cell detachment by scraping off caused a rapid reduction in Tyr phosphorylation of the 130 kDa protein (Fig 2B), as expected for p130cas. p130cas is also sensitive to the activity of protein tyrosine phosphatases [31], which can be inhibited by H_2_O_2_ [32]. We tested the effect of H_2_O_2_ treatment on Tyr phosphorylation of the 130 kDa protein. The results (Fig 2C) show that addition of H_2_O_2_ to the cells caused a dramatic increase in Tyr phosphorylation of the 130 kDa protein. Thus, the 130 kDa protein exhibits both hallmarks of p130cas.

To further demonstrate that the 130 kDa protein is p130cas, we performed co-immunoprecipitation western blot assays. Cell extracts were incubated with 4G10 monoclonal antibody and immunoprecipitated proteins were fractionated and analyzed by western blot using anti-p130cas antibodies. Fig 2D shows that anti-p130cas antibody recognized the 130 kDa protein in whole cell extracts and after immunoprecipitation with 4G10 antibody (lanes 1 and 2). Non-specific IgG do not bind p130cas (lane 4). Together, this demonstrates that ouabain affects Tyr phosphorylation of p130cas, but it remained to be investigated if the effect is direct, indirect or a combination.

### Src affects Tyr phosphorylation of the 130 kDa protein

**Fig 3:**
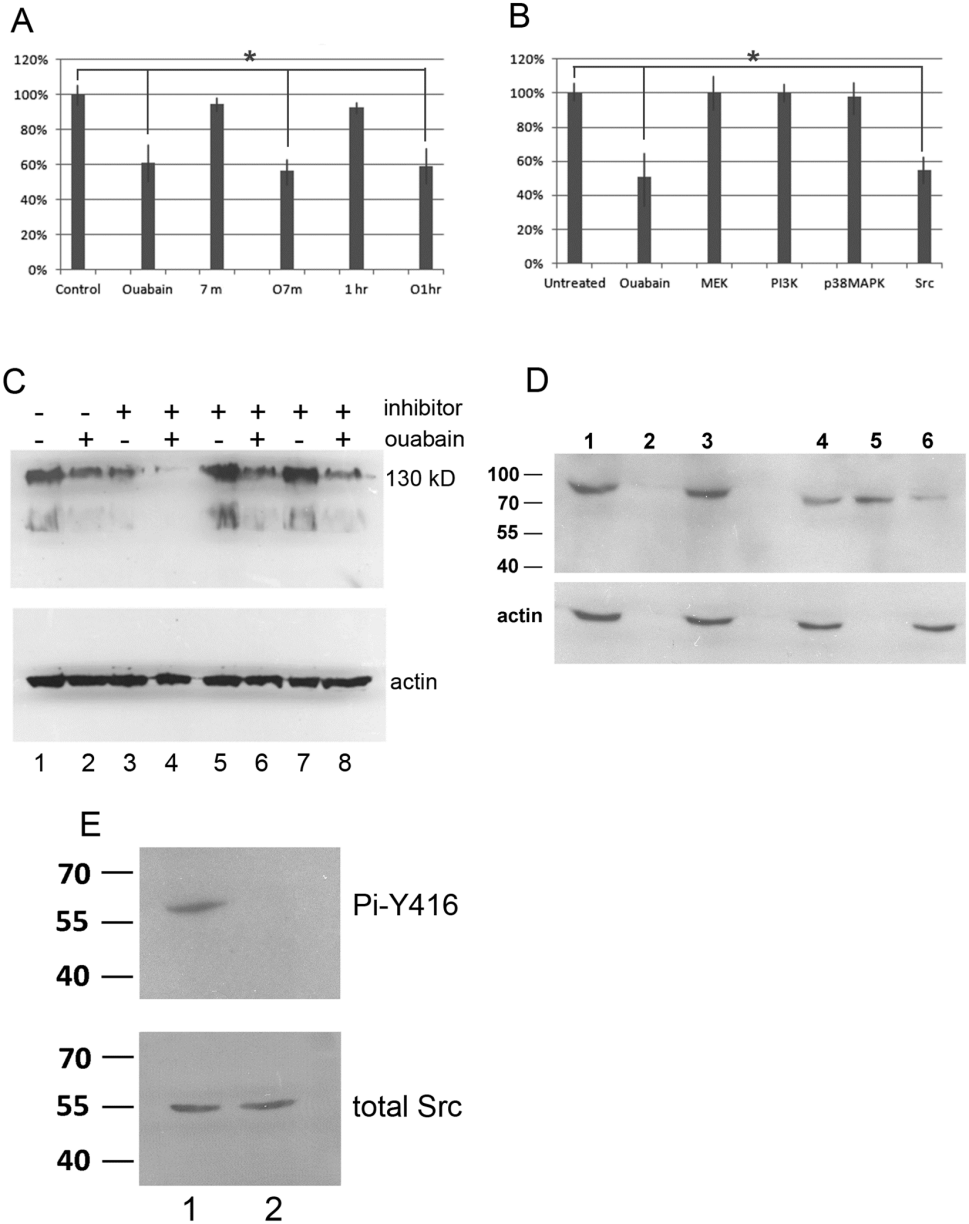
Src is involved in phosphorylation of p130cas. (A) Serum growth factors do not affect the phosphorylation status of p130cas. Cells were serum starved (in the presence or absence of ouabain) for 24 hrs after which serum was added for indicated times. Cell extracts were prepared at 7 min and 1 hr after serum addition, and analyzed by western blotting using 4G10 monoclonal antibody. The level of tyrosine phosphorylation of p130cas was measured using Image J. Control, untreated starved cells; Ouabain, starved cells pretreated with 200 nM ouabain; 7m, serum added for 7 min to untreated starved cells; O7m, serum added for 7 min to starved cells pretreated with ouabain; 1 hr, serum added for 1 hr to untreated starved cells; O1h, serum added for 1 hr cells to starved cells pretreated with ouabain. The level of tyrosine phosphorylation of p130cas in untreated cells was set at 100%. Quantitation was used to calculate statistically significant differences between groups, indicated with a * (P<0.05). (B) To analyze an effect of protein kinases on phosphorylation of p130cas, cells were treated with 500 nM ouabain or with 5 μM of inhibitors of the indicated protein kinases for 24 hrs, proteins extracted and the level of tyrosine phosphorylation of p130cas was measured as described above. The inhibitors used were: Src inhibitor AZD05030, MEK inhibitor PD0325901, PI3K inhibitor TGX221 and p38MAPK inhibitor VX702. The level of tyrosine phosphorylation of p130cas was set at 100% for untreated cells. Quantitation was used to calculate statistically significant differences between groups, indicated with a * (P<0.05). (C) Western blot analysis of cells treated with combinations of 500 nM ouabain and protein kinase inhibitors for 24 hrs. Upper panel: blots were probed with 4G10 monoclonal antibody; lower panel, blots were probed with anti-actin antibody. Cells were untreated (lane 1) or treated with ouabain (lane 2), Src inhibitor AZD05030 (lane 3), ouabain plus Src inhibitor AZD05030 (lane 4), MEK inhibitor PD0325901 (lane 5), ouabain plus MEK inhibitor PD0325901 (lane 6), p38MAPK inhibitor VX702 (lane 7), and ouabain plus p38MAPK inhibitor VX702 (lane 8). (D) To detect Src-Na,K-ATPase interaction, cells were ouabain treated (200 nM for 48 hrs) and extracts were analyzed by western blotting with anti- Na,K-ATPase antibody directly (lanes 1 and 4) or after immunoprecipitation using non-specific mouse IgG antibody (lanes 2 and 3) or anti-Src monoclonal antibody (lanes 5 and 6). Upper panel: lane 1, whole extract; lane 2, Na,K-ATPase bound to non-specific mouse IgG; lane 3, Na,K-ATPase not bound to non-specific mouse IgG; lane 4, whole extract; lane 5, Na,K-ATPase bound to Src; lane 6, Na,K-ATPase not bound to Src. The lower panel shows western blot controls using anti-actin antibody. (E) Western blot analysis of Src in untreated cells (lane 1) and cells treated with ouabain (200 nM for 48 hrs) (lane 2). Top panel (Pi-Y416), western blotting using anti-phospho tyrosine-416 Src antibody. Lower panel (total Src), western blotting using anti-Src antibody to detect total Src protein.

Na,K-ATPase has been shown to affect EGFR signaling [4,33]. To investigate signaling pathways that control Tyr phosphorylation of p130cas by Na,K-ATPase, we first tested whether p130cas phosphorylation responds to growth factor stimulation. RPE cultures were serum starved for one day, and then replenished with serum. p130cas Tyr phosphorylation was analyzed 7 min, 1 hr, and 2 hrs after addition of serum. Addition of serum did not affect p130cas Tyr phosphorylation (Fig 3A; compare Control, 7 m and 1 hr). Also, prior treatment of starved cells with ouabain reduced p130cas Tyr phosphorylation equally independent of time after serum addition (Fig 3A; compare Ouabain, O7m and O1h). This suggested that the observed p130cas Tyr phosphorylation did not involve EGFR.

We next tested the possible effect of inhibitors to other protein kinases, including Src inhibitor AZD05030 (IC50 = 3 nM), MEK inhibitor PD0325901 (IC50 = 0.33 nM), PI3K inhibitor TGX221 (IC50 = 5 nM) and p38MAPK inhibitor VX702 (IC50 = 3–20 nM). All inhibitors were used at 5 μM, corresponding to ~1000 fold IC50 values. RPE cells were incubated overnight in the presence of these inhibitors. Fig 3B shows that, with the exception of the Src inhibitor, none of the other inhibitors had a statistically significant effect on p130cas Tyr phosphorylation. The Src inhibitor resulted in a 45% reduction in p130cas Tyr phosphorylation similar to that achieved by ouabain treatment (Fig 3B).

To assess a possible connection between ouabain’s activity and Src, we co-incubated cells with ouabain and Src inhibitor AZD05030. p130cas Tyr phosphorylation was almost abolished using ouabain and AZD05030 together (Fig 3C; compare lane 4 to lanes 2 and 3). In contrast, no additive effect on p130cas Tyr phosphorylation was observed using ouabain and inhibitors for MEK or p38MAPK (Fig 3C, lanes 6 and 8), as expected from the results in Fig 3A. Another Src inhibitor PP2 gave results similar to the one observed for AZD05030 (not shown). This prompted us to analyze the possibility that ouabain may affect Src activity, which may be required for normal p130cas Tyr phosphorylation.

To investigate this, we tested if Na,K-ATPase binds to Src in ouabain-treated RPE cells using coimmunoprecipitation-western blotting. As shown in Fig 3D (top panel) Na,K-ATPase does not bind to non-specific mouse IgG (lane 2) and instead remains unbound (lane 3). However, when ouabain-treated cell extracts were incubated with Src antibody, Na,K-ATPase was immunoprecipitated by Src antibody (lanes 5 and 6 show Src-bound and unbound Na,K-ATPase, respectively). The lower panel shows actin western blot controls of the fractions analyzed in the top panel. We next analyzed the phosphorylation status of the Src Y416 residue, a well-documented indicator of Src activity, using an antibody against Src phospho-tyrosine 416. The results shown in Fig 3E indicate that, while ouabain treatment did not cause a change in the total amount of Src (bottom panel), ouabain caused a large reduction of Src Y416 phosphorylation (top panel). Our results suggest that ouabain enhances binding of Na,K-ATPase to Src, which binding may have affected Src kinase activity, which may account for the observed decrease in p130casTyr phosphorylation.

### Changes in p130cas affect cell migration

**Fig 4:**
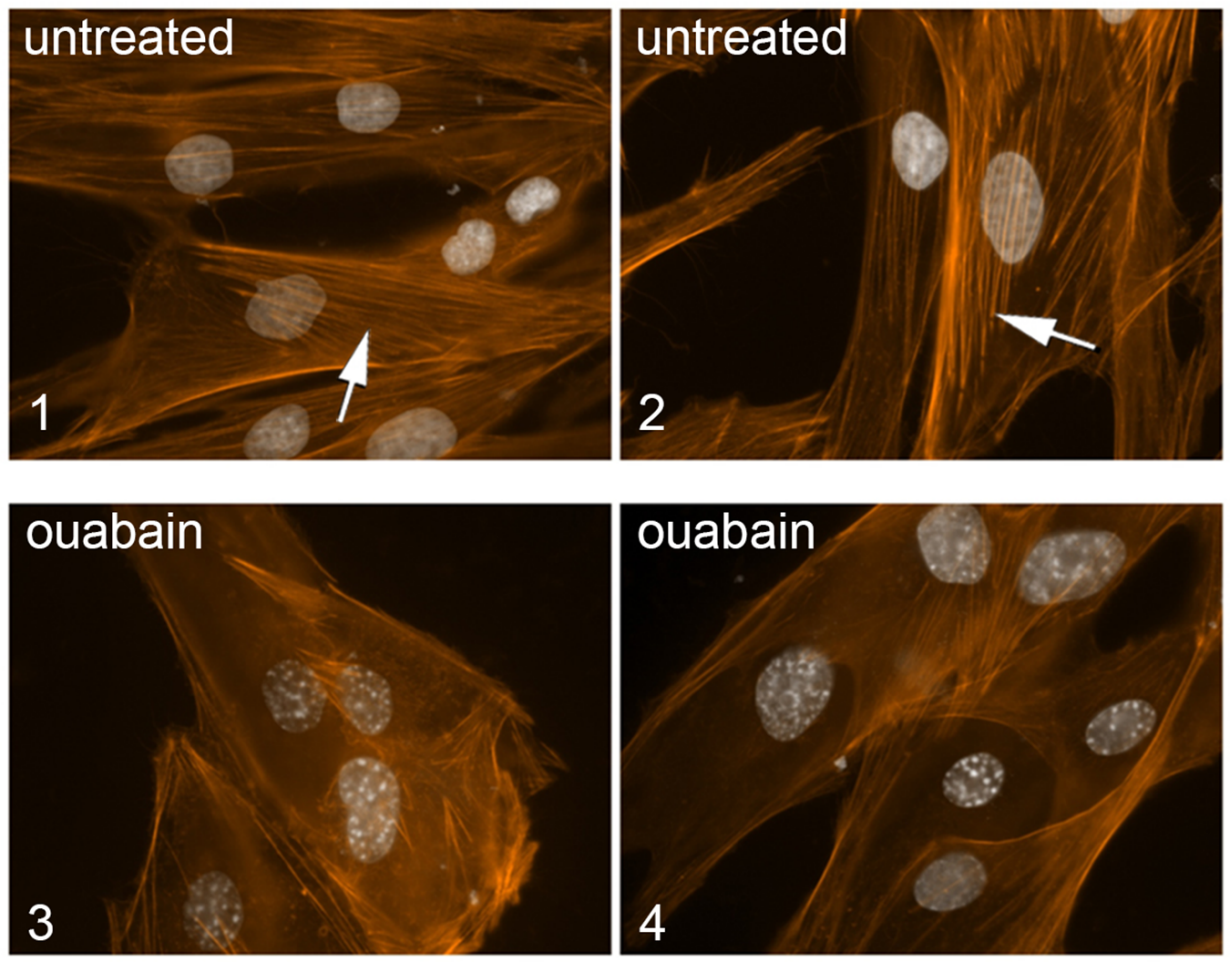
Ouabain affects actin polymerization. To analyze the effect of ouabain on actin polymerization we analyzed actin stress fibers as follows. Primary fibroblasts (HS68) were untreated, or treated with 50 nM ouabain for 24 hrs, fixed and stained with phalloidin. Panels 1 and 2, examples of untreated cells; panel 3 and 4, examples of cells treated with ouabain, showing a loss of stress fibers. Arrows point to stress fibers.

As mentioned, p130cas is a signaling node implicated in a variety of biological processes. In particular, actin stress fiber formation and cell migration are severely impaired in cells lacking p130cas [17,18]. Our finding that Na,K-ATPase regulates p130cas Tyr phosphorylation, predicted that ouabain treatment should affect actin polymerization. To study this, we analyzed untreated and ouabain-treated HS68 primary human foreskin fibroblasts for actin fibers using phalloidin. As predicted, fibroblasts treated with very low concentrations of ouabain (50 nM) displayed reduced actin stress fibers compared to controls (Fig 4, panels 1 and 2 show untreated controls, and panels 3 and 4 show examples of ouabain-treated HS68 cells), suggesting that ouabain, through Na,K-ATP, may impact cell motility.

**Fig 5:**
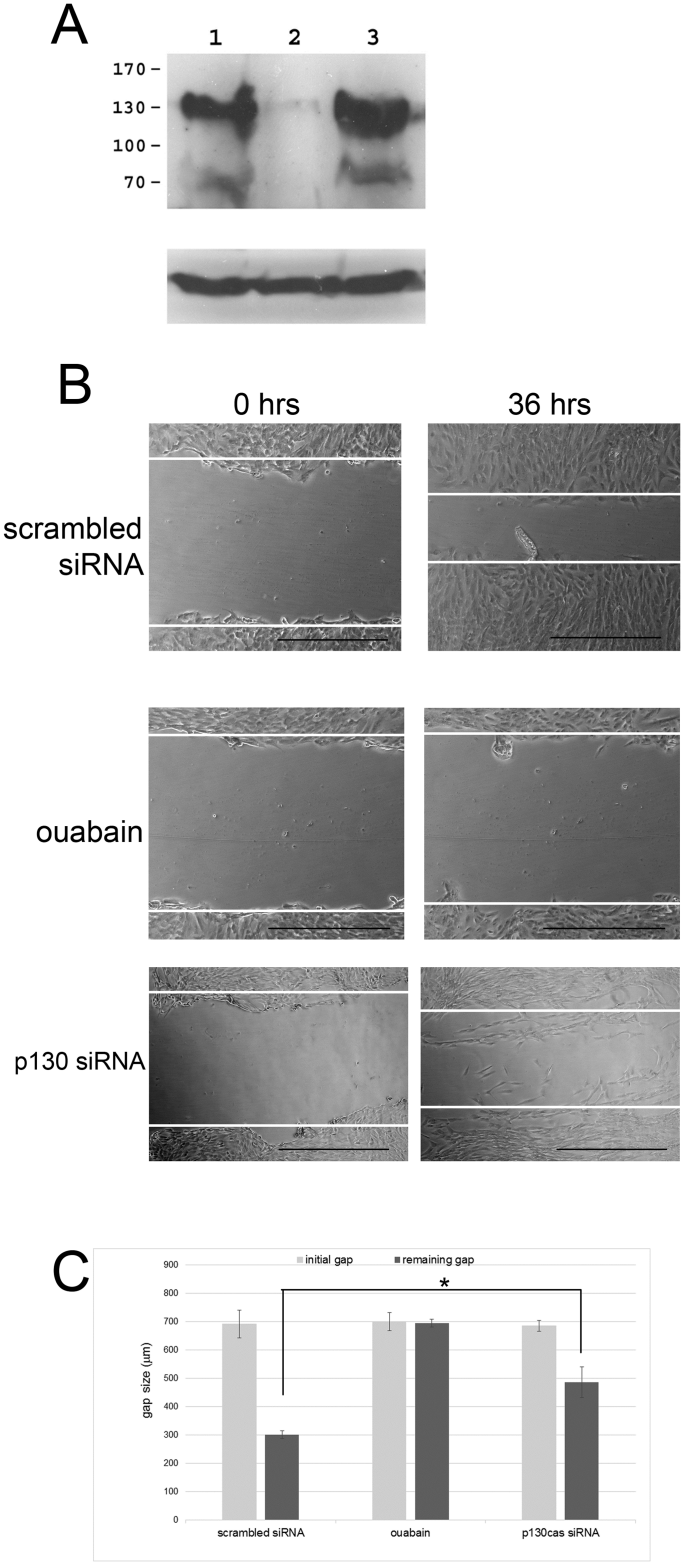
Ouabain regulates cell migration via p130cas. To analyze the effect of ouabain and the involvement of p130cas on cell migration cell scratch assays were carried out. (A) The efficacy of p130cas knock down by specific siRNA was analyzed by western blot analysis. Cells were untreated (lane 1) or transfected with siRNA specific for p130cas (lane 2) or a control scrambled siRNA (lane 3), and Tyr-phosphorylated p130cas protein levels were analyzed by western blotting using monoclonal antibody 4G10. Upper panel, western blot with 4G10 antibody; lower panel, western blot with anti-actin antibody. (B) Cell migration was analyzed using the cell scratch assay on confluent cultures of RPE cells. After applying a scratch, phase-contrast images were recorded immediately (0 hrs) to measure the extent of the original gap and after 36 hrs of incubation to measure the gap remaining after cell migration. Images were taken at 20x magnification. Scrambled siRNA, control condition of cells treated with a scrambled siRNA; ouabain, cells were treated with ouabain; p130cas siRNA, cells were treated with p130cas-speific siRNA. The bar denotes 500 μm. (C) Quantitation of cell migration results in the scratch assay. Data obtained from assays as shown in panel A were quantitated. The sizes of the initial gaps caused by the scratches (initial gap) and the sizes of gaps remaining after 36 hrs (remaining gap) were measured and are shown in μm. The initial gaps were approximately 700 μm in size. Ouabain blocked virtually all cell migration. The average remaining gap size was significantly different between control cells (300 μm) and p130cas siRNA-treated cells (480 μm) (P<0.05; indicated by *).

We therefore analyzed if modulation of total p130cas using siRNA and of p130cas Tyr phosphorylation by ouabain affects cell motility. To measure this, we performed monolayer cell scratch damage assays, commonly used for monitoring cell migration [22], that we had employed previously [19]. RPE monolayers were scratched, cells were allowed to migrate into the gap in the presence or absence of ouabain, and the gap was measured after the scratch was made (0 hrs) and after 36 hrs. We carried out scratch assays using cells transfected with p130cas-specific siRNAs as well as under control conditions. First, we used western blot analysis to show that p130cas-specific siRNAs caused a 90% reduction of p130cas in RPE cells (Fig 5A). Next, the scratch assay showed that p130cas-specific siRNAs reduced migration of cells in comparison to controls (see panels labeled “p130 siRNA” in Fig 5B). In comparison, cell migration is apparent in control experiments (compare gap width at 0 and 36 hrs in panels labelled “scrambled siRNA”). At 50 nM, ouabain abolished cell migration (compare panels labeled “ouabain” at 0 and 36 hrs). Results were quantitated (Fig 5C) and show that in comparison to control cells (normal migration, cells fill gap), p130cas-specific siRNA caused a significant decrease in migration (large remaining gap, with some cells migrating into the gap). The ouabain results were confirmed in Transwell assays: we observed that over a 24-hour period >500 RPE cells migrated through the Transwell membrane, but in the presence of 50 nM ouabain fewer than 5 migrated (not shown).

Together, these results demonstrate that ouabain changes p130cas Tyr phosphorylation, changes Na,K-ATPase/Src interaction, diminishes actin polymerization and ultimately inhibits cell migration.

### Ouabain causes nucleus-centrosome separation

**Fig 6:**
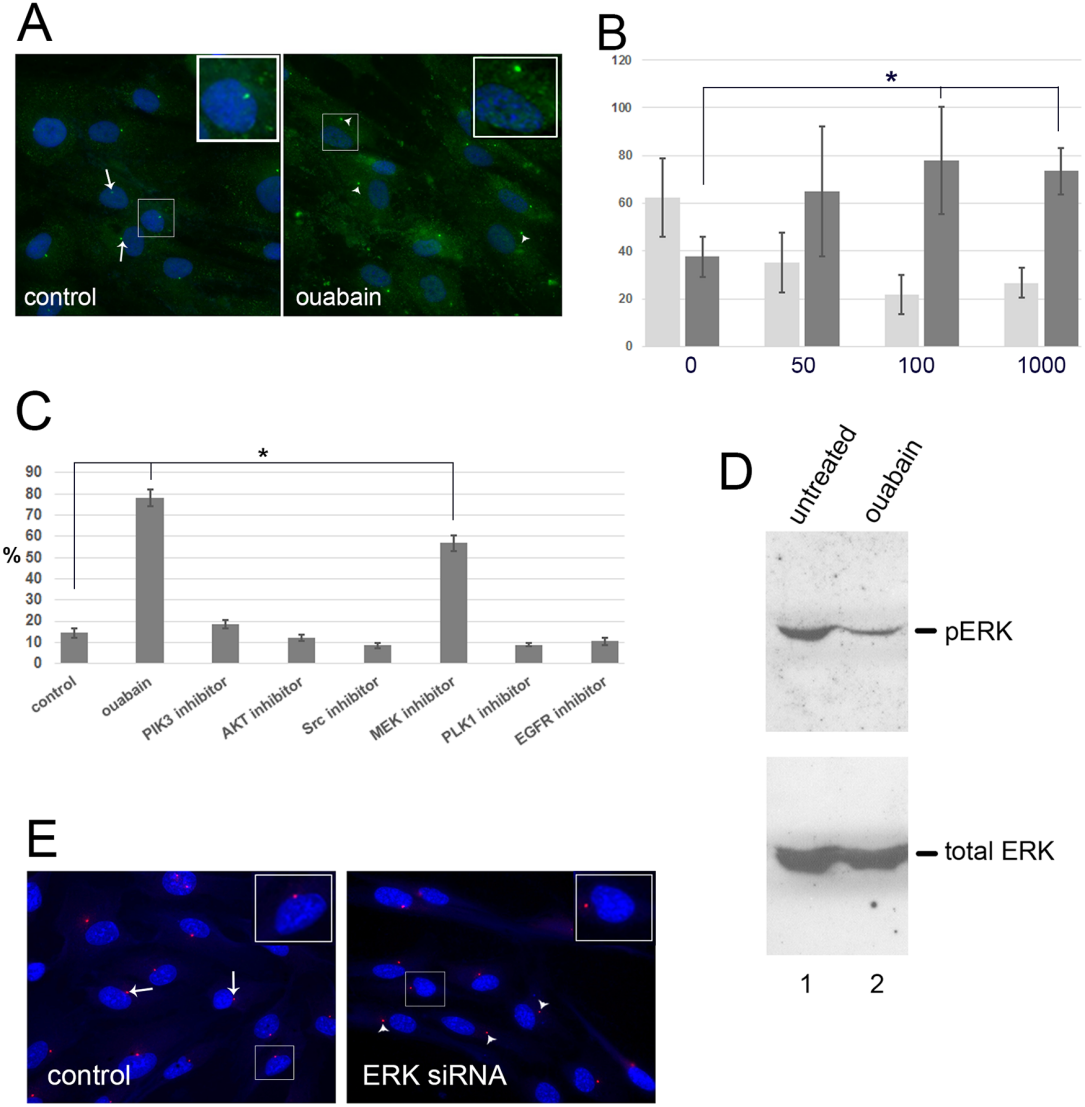
Ouabain inactivates ERK1,2 and causes nucleus-centrosome separation. (A) RPE cells were untreated (control) or treated with 100 nM ouabain (ouabain), fixed and processed for immunofluorescence microscopy. Centrosomes were visualized by staining using anti-ninein antibody, and DNA was visualized using DAPI. Arrows show examples of centrosomes close to the nucleus (control panel). Arrowheads point to examples of centrosomes that are separated from the nucleus (ouabain panel). Insets show enlarged area. (B) Assays shown in A were carried out and distances between nucleus and centrosomes measured to quantitate the percentage of cells showing separation of nucleus and centrosomes. Cells were untreated (0) or treated with 50, 100 and 1000 nM ouabain as indicated. Light grey bars represent the % of cells showing close association between nucleus and centrosome, and dark grey bars represent the % cells showing separated nucleus and centrosome. The difference between untreated (0 nM) and cells treated with 100 and 1000 nM ouabain is significant (P<0.05, indicated by *). (C) To analyze the possible involvement of kinases in the ouabain-induced separation of nucleus and centrosome, distances between nucleus and centrosome were measured in untreated cells (control) and in cells treated with ouabain (ouabain) or with the indicated kinase inhibitors. Quantitation was used to calculate the percentage of cells showing separation. Both ouabain treatment and the MEK inhibitor induced separation significantly different from control (P<0.05, indicated by *). (D) The effect of ouabain treatment on ERK1,2 phosphorylation was measured by western blotting using phosphor-ERK1,2-specific antibodies and total ERK1,2 antibodies. Cells were untreated (lane 1), or treated with 100 nM ouabain (lane 2). (E) To directly investigate the role of ERK1,2 in nucleus-centrosome separation we compared untreated cells (control) to cells treated with ERK1,2-specific siRNAs (ERK siRNA). Centrosomes were visualized by staining using anti-pericentrin antibody, and DNA was visualized using DAPI. Arrows point to examples of nucleus-centrosome arrangement in control cells. Arrowheads show examples of separated centrosomes in siRNA-treated cells. Insets show enlarged area.

While we were performing the above experiments which demonstrate that ouabain inhibits cell migration via p130cas, we were engaged in another line of investigation that showed that nucleus-centrosome separation can be induced by ATP resulting in inhibition of cell migration [19]. This suggested the possibility that ouabain’s effect on cell migration could involve nucleus-centrosome separation. This possibility was investigated using RPE cells. Fig 6A shows that treatment of RPE cells with ouabain indeed causes a separation of nucleus and centrosome.

Quantitation of such experiments, including dose-response assays, is shown in Fig 6B. These demonstrate that 50 nM and 100 nM ouabain cause an increased percentage of cells that show separation between the two organelles (dark grey bars) with a corresponding drop in the percentage of cells with the two organelles in close association (light grey bars). Doses higher than 100 nM do not further increase this percentage (Fig 6B). We next sought to identify the pathway involved in this process with a focus on kinase pathways, including Src. Cells were either untreated, treated with ouabain, or treated with inhibitors to the following kinases: PI3K, AKT, Src, MEK, Plk1 and EGFR and the percentage of cells with separated nucleus-centrosome was quantitated as done previously [19]. This shows that only the MEK inhibitor caused a significant percentage of cells to have separation of nucleus and centrosome. None of the other inhibitors tested, including the Src inhibitor, had a significant effect on nucleus-centrosome arrangement (Fig 6C). A combination of ouabain and MEK inhibitor treatments did not further change the % cells with nucleus-centrosome significantly (not shown). In Fig 3B we had demonstrated that Tyr-phosphorylation of p130cas is reduced by the Src inhibitor, but not by the MEK inhibitor. Together these results present the possibility (see Discussion) that ouabain induces two pathways ultimately blocking cell migration: the pathway involving p130cas/Src and the pathway involving MEK/nucleus-centrosome separation. To prove that ERK1,2 is involved in the ouabain effect we first analyzed by western blotting the phosphorylation status of ERK1,2 in untreated and ouabain-treated cells. Fig 6D shows a clear reduction in ERK phosphorylation upon ouabain treatment, indicative of loss of activity of ERK1,2. Ouabain did not affect total ERK1,2 levels. We next studied if ERK1,2 is critical for the normal close arrangement of the nucleus and centrosome by treating cells with ERK1,2-specific siRNAs. Fig 6E shows that control scrambled siRNA did not affect the arrangement as expected. However, ERK-siRNA caused nucleus-centrosome separation. Together these results show for the first time that ouabain affects nucleus-centrosome arrangement and that ERK is involved in this effect.

## Discussion

Na,K-ATPase is an essential enzyme that is estimated to consume ~25% of the entire ATP pool within the body to maintain sodium and potassium gradients across cell membranes, which are essential for the function and viability of all mammalian cells [34]. One pivotal regulator of this enzyme is ouabain, which is present in human plasma [35,36]. The level of ouabain is in the picomolar-nanomolar range and fluctuates with physiological conditions such as exercise, meals and pregnancy [37]. In addition to human sera, ouabain was also detected in cerebral spinal fluid, as well as in commercial bovine and horse sera [38]. Altered levels of the ouabain analogue were detected in patients with arterial hypertension, eclampsia and myocardial infarction [35,39]. These data suggest that ouabain functions as an endogenous hormone [12,40].

Here we investigated the possible mechanisms involved in the negative effect of ouabain on cell migration. It has been well established that -in addition to the critical pump activity- Na,K-ATPase nanomolar concentrations of ouabain activate its function as a signal transducer implicated in a number of signaling pathways [4–6,41]. We discovered that nanomolar concentrations of ouabain affect Tyr phosphorylation of p130cas, a signal node implicated in many signaling pathways [42,43], and that this involves Src. We found that in RPE cells, at low concentrations, ouabain causes a loss of p130cas Tyr phosphorylation, enhanced binding of p130cas to Src and a reduction of Src Tyr phosphorylation at Y416, indicative of inhibition of Src activity. Src inhibition likely contributes to the observed reduction in p130cas Tyr phosphorylation, a well-known Src substrate [24,26], because we show that the use of Src kinase inhibitors -but not inhibitors to other kinases tested- also decreases Tyr phosphorylation of p130cas. This suggests that p130cas is a downstream target of Na,K-ATPase signalling. Ouabain/Na,K-ATPase signalling can however have different effects in different cell lines: ouabain was reported to activate Src and/or IP3R pathways resulting in Tyr phosphorylation of multiple proteins and consequently stimulating cell proliferation and viability [38,44–46], whereas other studies have concluded that ouabain, through Na,K-ATPase, inhibits Src and inhibits proliferation, e.g. of T lymphocytes (both CD4+ and CD8+) and peripheral blood mononuclear cells [47,48]. Thus the effect exerted by ouabain through the Na,K-ATPase protein may be concentration dependent, cell-specific, and/or linked to the particular signaling pathway activated [49]. Our result is consistent with recent work showing reduced Src activity after ouabain treatment of transformed A549 human cells [50].

We also found that a reduction in p130cas Tyr phosphorylation by ouabain causes loss of actin stress fibers and inhibits cell migration, again implicating p130cas as a Na,K-ATPase downstream target in these processes. It should be noted that inhibition of Src by ouabain/Na,K-ATPase may also affect other downstream substrates and pathways. This raises the question: does ouabain/Na,K-ATPase directly interact with p130cas or does it signal via Src? We show here that in RPE cells Na,K-ATPase interacts with Src, and earlier studies showed that Src binds p130cas. However, using co-immunoprecipitation-western blotting we failed to show direct interactions of Na,K-ATPase and p130cas (not shown). It should be noted that Focal adhesion kinase (FAK) is a substrate of Src [51,52] and forms a complex with p130cas [24,25]. Src-FAK-p130cas complexes can dissociate upon loss of cell attachment to the matrix, resulting in reduced phosphorylation of p130cas, and Na/K-ATPase appears close to this complex [53]. A possible role of FAK in the ouabain effect we document remains to be investigated.

In a previous study we had shown that ATP blocks cell migration and does so by inducing the Epac1/RapGef3 pathway in cells resulting in separation of nucleus and centrosome [19]. In this work, we addressed if ouabain can also affect nucleus-centrosome arrangement, in addition to its activation of the p130cas/Src pathway. We demonstrate for the first time that ouabain causes separation of nucleus and centrosome. Our analysis of pathways shows that the ERK1,2 kinase is directly involved. Indeed, ouabain decreases ERK1,2 phosphorylation, an indicator of reduced activity, and ERK1,2-specific siRNAs cause separation of the two organelles. ERK1,2 signalling is well documented and impacts many processes including cell proliferation and mitosis. Earlier studies have identified activated ERK1,2 on kinetochores and spindle poles [54] and on centrosomes [55]. Raf1 protein has also been localized to centrosomes [56] indicating the existence of an efficient Raf1-ERK1,2 signaling pathway to regulate mitosis via AuroraA kinase.

In comparison, little is known about a direct role for ERK1,2 in the coordination of nucleus and centrosome localization. We show that inhibitors of EGFR, PI3K, AKT, Plk1 and Src did not induce separation of nucleus and centrosome in RPE cells, whereas ERK1,2 inhibitors and siRNAs did. This suggested the possibility that ouabain/Na,K-ATPase activates ERK1,2 without involvement of the paradigm EGFR-Raf1-ERK1,2 signalling pathway. Indeed, our results thus suggest that the ouabain-activated p130cas/Src pathway may not be directly linked to the ERK1,2/nucleus-centrosome path. Further studies should reveal the players involved in the signalling from ouabain/Na,K-ATPase to ERK1,2. Intriguingly, we show internalization of ouabain at 30 hrs (Fig 1D), which process is already detectable at 7 hrs. A recent report describes that Na,K-ATPase bound to ouabain can be internalized possibly for lysosomal degradation [57]. We do not know if fluorescent-labeled ouabain is entering the cell free or -more likely- bound to Na,K-ATPase: in the latter case it is conceivable that this complex triggers signaling pathways involving ERK1,2 independent of EGFR or Src-mediated steps.

**Fig 7:**
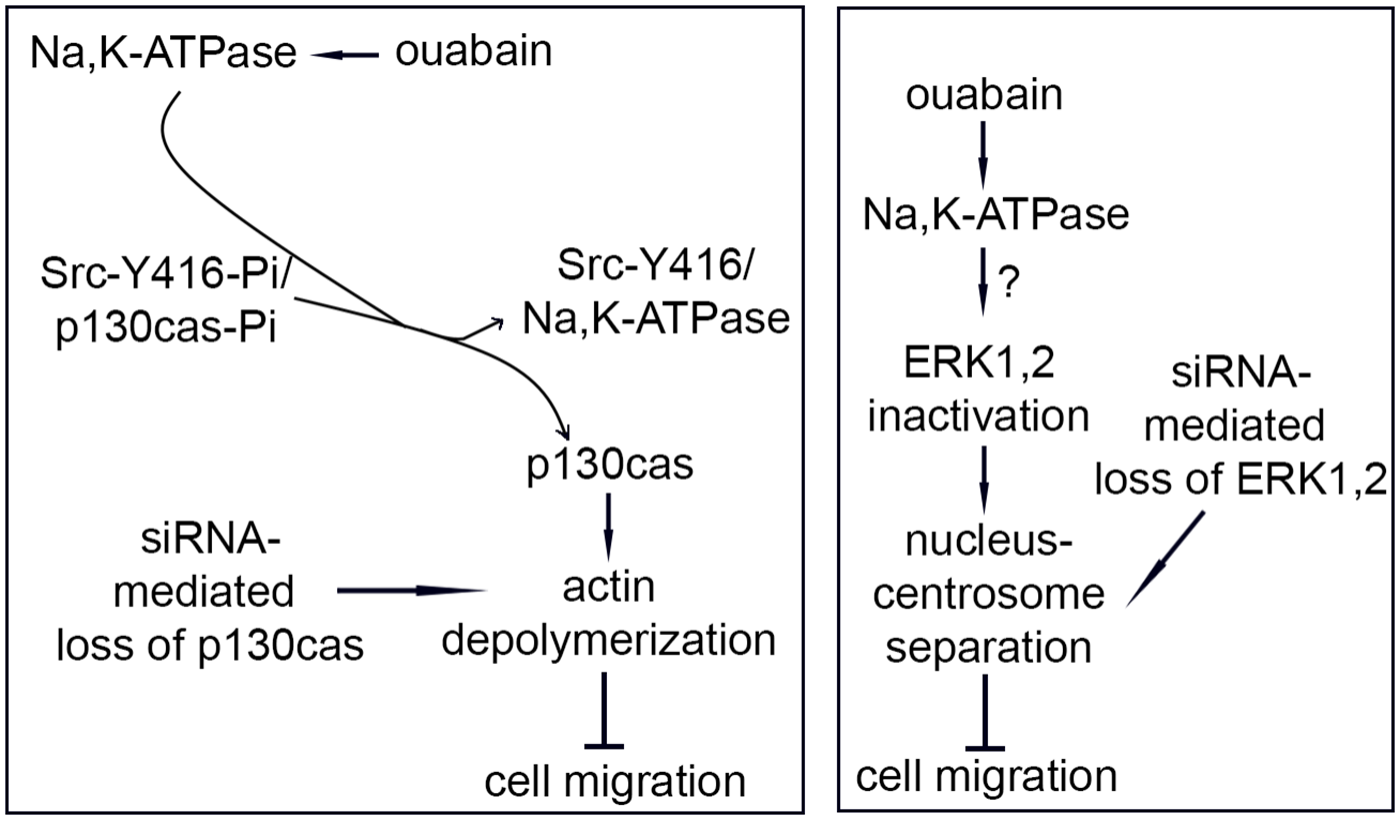
Models for ouabain action on cell migration. Two models are schematically presented that incorporate all results. Left panel: ouabain activates Na,K-ATPase, resulting in a shift in binding of Src and loss of the phosphor group on Src-Y416 and dephosphorylation of p130cas. Inactivation of p130cas or loss (via siRNA) results in loss of actin stress fibers and a block in cell migration. Right panel: ouabain activates Na,K-ATPase, resulting in inactivation of ERK1,2. The intermediate steps are unknown (indicated by?). ERK1,2 inactivation or loss (via siRNA) leads to nucleus-centrosome separation and a block in cell migration.

Our results are schematically represented in the models shown in Fig 7.

In summary, we identified two pathways for ouabain/Na,K-ATPase that regulate cell migration: one is via p130cas Tyr phosphorylation, directly impacting actin polymerization, and the second is via ERK1,2 causing nucleus centrosome separation.
